# The function of Drosophila larval class IV dendritic arborization sensory neurons in the larval-pupal transition is separable from their function in mechanical nociception responses

**DOI:** 10.1371/journal.pone.0184950

**Published:** 2017-09-14

**Authors:** Hannah E. Brown, Trishna Desai, Allison J. Murphy, Harshida Pancholi, Zachary W. Schmidt, Hannah Swahn, Eric C. Liebl

**Affiliations:** Department of Biology, Denison University, Granville, Ohio, United States of America; McGill University, CANADA

## Abstract

The sensory and physiological inputs which govern the larval-pupal transition in Drosophila, and the neuronal circuity that integrates them, are complex. Previous work from our laboratory identified a dosage-sensitive genetic interaction between the genes encoding the Rho-GEF Trio and the zinc-finger transcription factor Sequoia that interfered with the larval-pupal transition. Specifically, we reported heterozygous mutations in *sequoia* (*seq*) dominantly exacerbated the *trio* mutant phenotype, and this *seq*-enhanced *trio* mutant genotype blocked the transition of third instar larvae from foragers to wanderers, a requisite behavioral transition prior to pupation. In this work, we use the GAL4-UAS system to rescue this phenotype by tissue-specific *trio* expression. We find that expressing *trio* in the class IV dendritic arborization (da) sensory neurons rescues the larval-pupal transition, demonstrating the reliance of the larval-pupal transition on the integrity of these sensory neurons. As nociceptive responses also rely on the functionality of the class IV da neurons, we test mechanical nociceptive responses in our mutant and rescued larvae and find that mechanical nociception is separable from the ability to undergo the larval-pupal transition. This demonstrates for the first time that the roles of the class IV da neurons in governing two critical larval behaviors, the larval-pupal transition and mechanical nociception, are functionally separable from each other.

## Introduction

Understanding the molecular basis of behavior is a broad, overarching goal in neurobiology. In the Drosophila model system, the mechanisms governing the transition from larvae to pupae have been investigated on multiple levels. Numerous physiological mechanisms have been identified as having a role in the larval-pupal transition by directly or indirectly regulating ecdysone production [1–6]. Neuronal mechanisms regulating the larval-pupal transition have also been defined. Jayakumar *et al*. identified a key neural circuit that detects external nutrient levels that in turn drive ecdysone production [7]. Wu *et al*. showed that down-regulation of neuropeptide F regulates the food aversion of larvae just prior to pupation [8] as they transition from feeding forager larvae to non-feeding wander larvae [9].

Specific sensory neurons critical for the larval-pupal transition have also been identified. The Drosophila larval dendritic arborization (da) sensory neurons are segmentally stereotyped neurons grouped into classes I through IV based on their characteristic dendritic morphologies, with class IV neurons having the most complex arbors [10]. *pickpocket* (*ppk*) was identified as encoding a Degenerin/Epithelial Sodium Channel (DEG/ENaC) subunit, and in third instar larvae *ppk* expression is confined to the class IV da neurons [11,12]. An exhaustive examination of the *ppk* mutant phenotype has revealed disruptions of larval crawling behavior, the length of the larval terminal growth period, and alterations in the timing of the larval-pupal transition, leading to the conclusion that sensory input from the Ppk-expressing neurons (the class IV da neurons) critically influences the larval-pupal transition [11,13,14].

Previous work from our laboratory identified a dosage-sensitive genetic interaction between the genes encoding the Rho-GEF Trio and the zinc-finger transcription factor Sequoia that interfered with the larval-pupal transition [15]. In genetic terms, we reported that a heterozygous mutation in *sequoia* (*seq*) dominantly enhanced the *trio* mutant phenotype, as animals simply heterozygous mutant for *seq* or simply homozygous mutant for *trio* were well-represented as pupae, but animals both heterozygous mutant for *seq* and homozygous mutant for *trio* were dramatically under-represented as pupae. However, those heterozygous *seq*, homozygous *trio* mutant animals were fully represented as third-instar larvae. Those larvae failed to pupate because they failed to undergo the transition from forager larvae to wanderer larvae [15].

In this work, we reasoned that testing for rescue to pupation by driving *trio* in tissue-specific patterns in the heterozygous *seq*, homozygous *trio* mutant background would define the larval tissues critically impacted by that mutant genotype. As both *seq* and *trio* have been functionally linked to dendrite morphology [16–18], and based on the demonstrated functionality of the class IV da neurons in the larval-pupal transition [11,13,14], we hypothesized that the class IV da neurons may play a critical role in our *seq*-enhanced *trio* mutant phenotype. Here we show that expressing *trio* exclusively in the class IV da neurons in our *seq*-enhanced *trio* mutant background can rescue the larval-pupal transition. Examining the class IV da neuron morphologies in wildtype, mutant and rescued animals shows that an over-branched dendritic arbor-phenotype correlates with the disruption of the larval-pupal transition.

Class IV da sensory neurons are also critical for nociception. Optogenetic stimulation of class IV da neurons evokes nocifensive responses [19]. Silencing class IV da neurons by tissue-specific tetanus toxin light chain-expression eliminates both thermal and mechanical nociception [19,20]. Similarly, disrupting Pickpocket activity in class IV da neurons by either classical genetic methods or RNAi expression decreases mechanical nociception responses [21]. Thus, as the class IV da neurons provide sensory input for nociceptive responses, we also tested wildtype, *seq*-enhanced *trio* mutant and rescued larvae for mechanical nociception and found that larval-pupal behavior is separable from mechanical nociception responses. Therefore, this work shows that the class IV da sensory neurons’ function in the larval-pupal transition is separable from their function in nociception responses.

## Materials and methods

### Genetics

#### Fly stocks and maintenance

All flies were maintained on either standard cornmeal-yeast medium or Formula 4–24 Instant Food (Carolina Biological) in humidified incubators at 25°C. *UAS-trio*.*B* (# 9513) and *GAL4-elav*.*L* (# 8765) were obtained from the Bloomington Drosophila Stock Center. The hypomorphic *trio* allele *trio*^*P1*^, also known as *P{lacW} trio*^*s036810*^ [22–24], was obtained from the Szeged Stock Center and is maintained in our laboratory. The *GAL4-ppk1*.*9*, *UAS-mCD8GFP* chromosome, which expresses GAL4 exclusively in the class IV da sensory neurons [11] and expresses a membrane-targeted Green Fluorescent Protein (GFP) construct [25], was a gift from Dr. Wesley Grueber. *seq*^*9*.*17*^ and *trio*^*M89*^ were generated in our laboratory and have been described previously [15,23].

#### Rescue of the *seq*-enhanced *trio* mutant phenotype

All crosses were done with both males and females carrying the *T(2;3) SM6a-TM6B* balancer chromosomes, which are marked with the dominant phenotypic markers *Tubby* (*Tb)* and *Curly* (*Cy*). The *UAS-trio*.*B* construct was recombined onto the *trio*^*P1*^ allele. *GAL4-elav*.*L; trio*^*M89*^ */ T(2;3) SM6a-TM6B* or *GAL4-ppk1*.*9*, *UAS-mCD8GFP; trio*^*M89*^ */ T(2;3) SM6a-TM6B* were crossed to *seq*^*9*.*17*^*; trio*^*P1*^, *UAS-trio*.*B / T(2;3) SM6a-TM6B*. Controls excluded a *GAL4* construct or the *seq*^*9*.*17*^ allele. Eight males and eight virginal females were crossed in individual vials and these were transferred to fresh vials every 24 hours. After nine days of development the pupae in each vial were scored using the *Tubby* marker. The counts from five broods from the same parental flies were combined as a single data point. As a Mendelian ratio of 2 Tubby: 1 non-Tubby (i.e. wildtype) pupae was expected in all cases, the number of non-Tubby pupae was divided by half the number of Tubby pupae to obtain the percent of expected pupae.

### Determining morphologies of the class IV da neurons

#### Image capture of the class IV da neurons

Twenty-five males of the genotype *GAL4-ppk1*.*9*, *UAS-mCD8GFP; trio*^*M89*^ */ T(2;3) SM6a-TM6B* were crossed to twenty-five virginal females of the appropriate genotype balanced over *T(2;3) SM6a-TM6B* in bottles containing Formula 4–24 Instant Food (Carolina Biological). These crosses were transferred to new bottles every 24 hours. 96–120 hour-old progeny larvae were recovered by flotation after re-suspending the food plug in 3M NaCl. The *Tubby* marker was used to score larvae, with non-Tubby (wildtype) larvae being the phenotype/genotype of interest. The larvae were immobilized using a similar technique to that described in Weiner *et al*. [26]. A platform was generated by gluing two 18mm x 18mm coverslips on top of each other onto a microscope slide. The larvae were placed on this platform. As they began to crawl normally (dorsal side up), they were immobilized under a 50mm x 22mm coverslip taped at both ends to the slide. The slight bowing of the 50mm cover slip across the larvae effectively trapped its epidermis; although the gut and internal musculature were often motile, the class IV da neurons were effectively immobilized for confocal microscopy.

A Nikon A1R Live Cell Imaging confocal microscope was used to image portions of two of the dorsal-most class IV neurons (the ddaC neurons) from cell body-to-cell body, on both sides of the dorsal midline, within any one segment from A1 to A5. Images were captured with 2x averaging. The field encompassing the two ddaC neurons’ cell bodies was typically 300–350μm x 600–750μm.

#### Image analysis

Maximum intensity projections were generated from the appropriate confocal stacks using ImageJ [27] and saved as tiff files. These were analyzed with Imaris’ filament tracer package (Bitplane) to generate total dendrite lengths and total branch points. As all our images contained dendritic filaments that entered the image from the periphery, and were therefore not connected within the image to either of the ddaC cell bodies, these were blacked out using ImageJ’s paintbrush tool before Imaris analysis. If this was not done, Imaris’ filament tracer added in false connecting lines, distorting the calculated dendrite lengths and branch point counts. These peripheral dendritic filaments were analyzed (filament length and branch points) by hand using ImageJ. Iso-neuronal avoidance defects and hetero-neuronal tiling defects were scored by hand.

Total dendritic lengths were normalized to the area of the field analyzed. Total dendritic branches were normalized to the total dendritic length within the field analyzed. Iso-neuronal avoidance defects were normalized to the total dendritic length within the field analyzed. Hetero-neuronal tiling defects were normalized to the length of the path defining the interface between the two ddaC dendritic fields.

### Mechanical nociception assays

Non-*Tubby* (wildtype) 96–120 hour-old larvae of the appropriate genotype were generated exactly as described above for image capture of the class IV da neurons. 50 mN Von Frey filaments were made, and larvae were tested in an arena with conditioned water as described [19,21]. Under a dissecting microscope, individual larvae were stimulated with the Von Frey filament on their dorsal side between segments A4 and A6. If the larvae executed a 360-degree nocifensive rolling response [19,28], it was scored as a positive response. If the larvae did not execute a full-360-degree roll, it was stimulated a second and if needed a third time. Larvae that did not respond after the third stimulation were scored as non-responsive.

### Statistical analyses

Data were analyzed using the JMP Pro 12 statistical software package (SAS Institute) and consisted of MANOVA, one-way ANOVA followed by Tukey-Kramer HSD pairwise analyses and Chi-Squared analysis.

## Results and discussion

### Tissue-specific expression of *trio* in the class IV da sensory neurons rescues the *seq*-enhanced *trio* mutant phenotype

**Fig 1:**
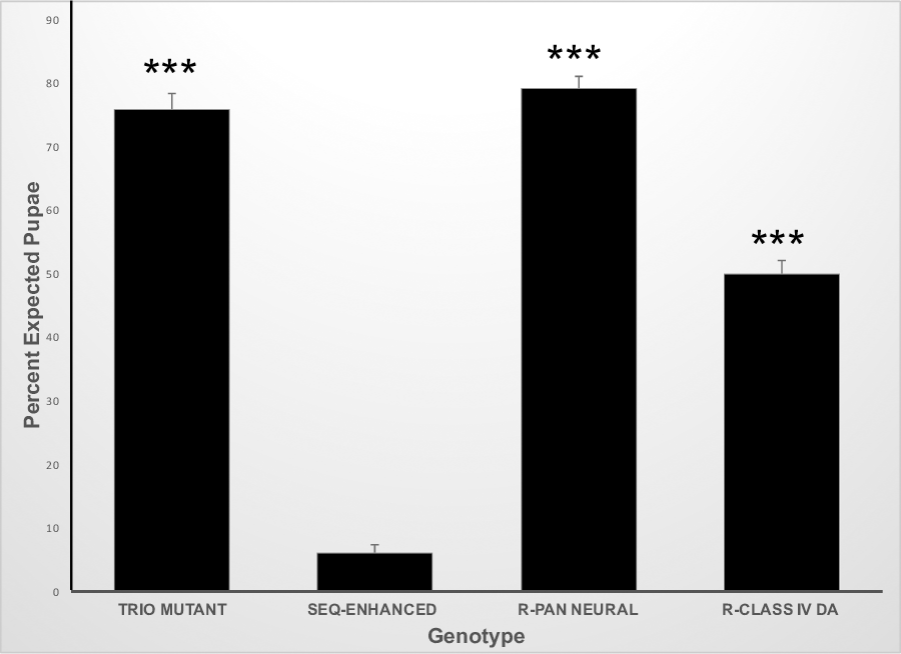
Tissue-specific expression of *trio* rescues pupation in the *seq*-enhanced *trio* mutant background. The percent of expected pupae was determined for the following genotypes: *trio*-mutant, *trio*-mutant background *(trio*^*M89*^ */ trio*^*P1*^*); seq*-enhanced, *seq*-enhanced *trio* mutants (*seq*^*9*.*17*^ */ +; trio*^*M89*^ */ trio*^*P1*^*);* R-pan neural, rescue by pan-neural *trio* expression in the *seq*-enhanced *trio* mutant background (*GAL4-elav*.*L* / *seq*^*9*.*17*^*; trio*^*M89*^ */ trio*^*P1*^, *UAS-trio*.*B*); R-class IV da, rescue by class IV da *trio* expression in the *seq*-enhanced *trio* mutant background (*GAL4-ppk1*.*9*, *UAS-mCD8GFP* / *seq*^*9*.*17*^*; trio*^*M89*^ */ trio*^*P1*^, *UAS-trio*.*B)*. N = 15 for all genotypes. ANOVA p-value <0.0001; *** indicates pair-wise statistical significance at p<0.001 as compared to *seq*-enhanced. R-pan neural and R-class IV da are also statistically different from each other at p<0.001. Error bars represent the standard error of the means.

We had previously shown that the *seq*^*9*.*17*^ allele acted as a dominant enhancer of a sensitized *trio* mutant background, blocking the larval-pupal transition [15]. This observation has allowed us to identify the tissue responsible for this phenotype by rescuing pupation via driving *trio* in various tissue-specific patterns with the GAL4-UAS system [29]. In control crosses, we recovered 75.8±2.6% of the expected pupae of the simple *trio* mutant genotype, while we recovered only 6.1±1.1% of the expected pupae of the *seq*-enhanced *trio* mutant genotype (Fig 1). Driving *trio* with the *GAL4-elav*.*L* pan-neural driver in this *seq*-enhanced *trio* mutant background rescued pupation (79.2±1.8% expected pupae, Fig 1). Driving *trio* specifically in the class IV da sensory neurons with the *GAL4-ppk1*.*9* driver in this *seq*-enhanced *trio* mutant background also rescued pupation (49.9±2.2% expected pupae; Fig 1), however, the magnitude of this rescue was statistically lower than that achieved by pan-neural *trio* expression.

Rescue of pupation by driving *trio* expression with the *GAL4-elav*.*L* pan-neural driver (Fig 1) shows the perturbation of the larval-pupal transition is due to a correctable defect within the general larval nervous system. Rescue of pupation by driving *trio* with the *GAL4-ppk1*.*9* driver (Fig 1) shows the perturbation of the larval-pupal transition is due to a correctable defect within the larval class IV da sensory neurons specifically. The lower rescue provided by the *GAL4-ppk1*.*9* driver could be due to differences in the timing and/or level of expression within the class IV neurons between the two drivers, or it could indicate there are other sensory networks involved.

### Class IV da sensory neuron morphologies differ between genotypes

As *trio* encodes a Rho-GEF [23,24,30], we hypothesized that its impact on class IV da sensory neuron function could be through an impact on the morphology of these neurons. Thus, we carefully examined the morphologies of representative class IV da sensory neurons in our various genetic backgrounds.

**Fig 2:**
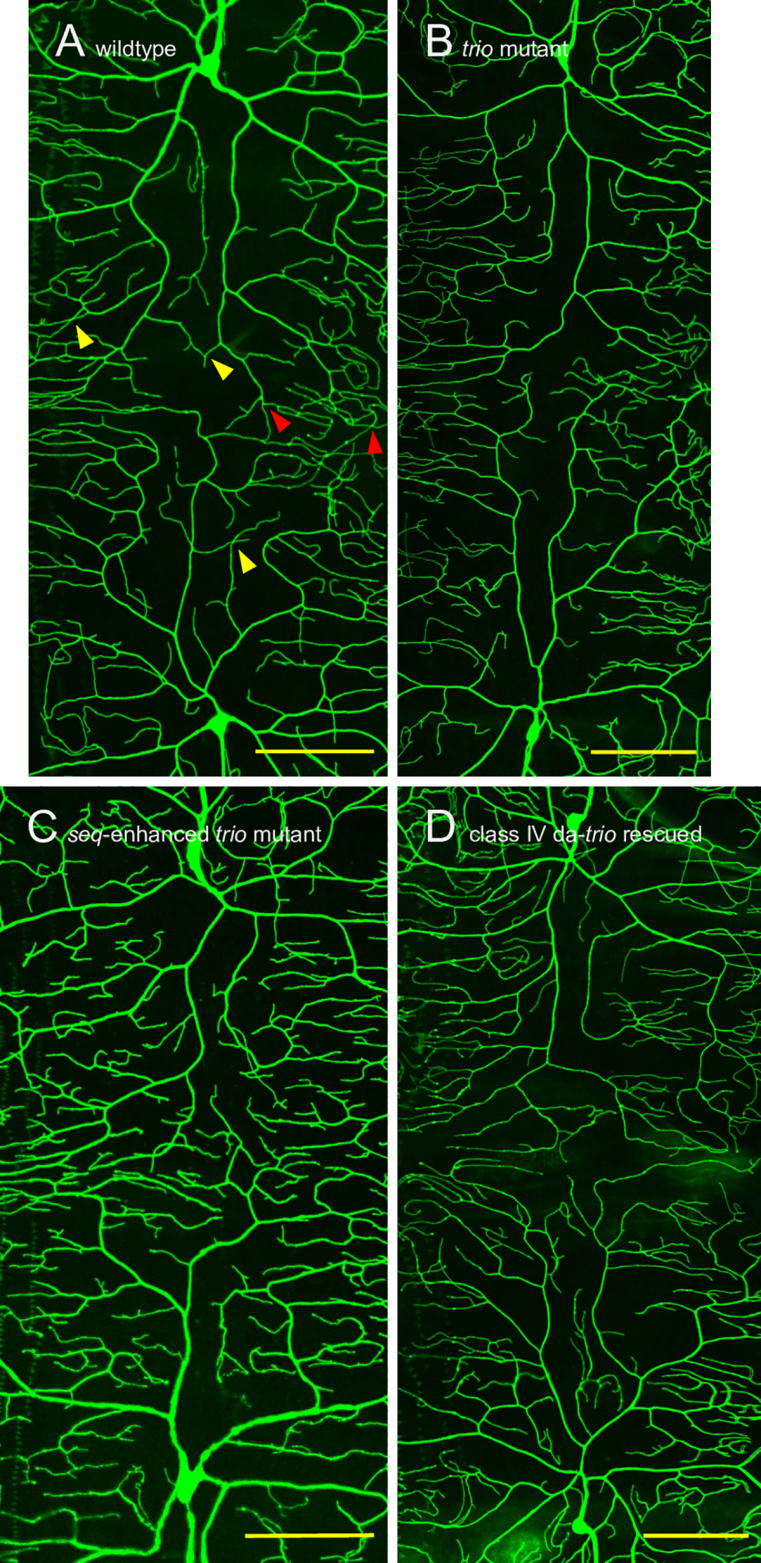
Representative ddaC morphologies in control, mutant and rescued third instar larvae. (A) ddaC in wildtype larvae (*GAL4-ppk1*.*9*, *UAS-mCD8GFP / +; trio*^*M89*^ */ +*, image shown is 300.8 μm x 650.5μm). (B) ddaC in simple *trio* mutant larvae (*GAL4-ppk1*.*9*, *UAS-mCD8GFP* / +; *trio*^*M89*^ */ trio*^*P1*^, image shown is 302.8 μm x 749.8μm). (C) ddaC in *seq*-enhanced *trio* mutants (*GAL4-ppk1*.*9*, *UAS-mCD8GFP* / *seq*^*9*.*17*^; *trio*^*M89*^ */ trio*^*P1*^, image shown is 300.4 μm x 601.7μm). (D) ddaC in *ppk1*.*9-trio* rescued *seq*-enhanced *trio* mutant larvae (*GAL4-ppk1*.*9*, *UAS-mCD8GFP* / *seq*^*9*.*17*^*; trio*^*M89*^ */ trio*^*P1*^, *UAS-trio*.*B*, image shown is 351.1 μm x 751.2μm). The dorsal midline runs horizontally through the approximate center of each image. In (A) representative examples of iso-neuronal avoidance defects are indicated with yellow triangles while representative examples of hetero-neuronal tiling defects are indicated with red triangles. Scale bars show 100 μm.

Utilizing the *GAL4-ppk1*.*9*, *UAS-mCD8GFP* chromosome, which expresses class IV da-specific membrane-targeted GFP, we characterized morphologies of the dendritic fields extending from a ddaC (the dorsal-most class IV da neuron) cell body, across the dorsal midline, to the mirror-image ddaC cell body within individual segments. This was done in wildtype larvae (Fig 2A), simple *trio* mutants (Fig 2B), *seq*-enhanced *trio* mutants (Fig 2C), and *ppk1*.*9-trio* rescued *seq*-enhanced *trio* larvae (Fig 2D). We were unable to use this technique to characterize these ddaC dendritic morphologies in the *GAL4-elav*.*L; UAS-trio*.*B* rescued larvae, as the *elav*.*L* driver would cause pan-neural GFP expression. Similarly, we were unable to characterize axonal morphologies with this technique, as the fasciculation of the class IV da neurons’ axons prevented the analysis.

Across our control, mutant and rescued genotypes we quantified four separate measures of ddaC dendritic morphology: dendritic density (dendrite length in μm normalized to μm^2^ surface), dendritic branch frequency (dendrite branches normalized to total dendrite length in μm), iso-neuronal avoidance defects (inappropriate dendrite crossings within a neuron’s field normalized to total dendrite length) and hetero-neuronal tiling defects (inappropriate dendrite crossings between two neurons’ fields normalized to the path length of the interface between the two dendritic fields). We used a MANOVA to examine if genotype affected the combined measures of dendritic morphology and found strong differences (Wilk's Lambda = 0.32453, approximate *F* = 3.40_12,55.02_, *p*<0.005). We thus did univariate analyses to determine which dependent variables were contributing to the differences amongst genotypes.

#### Dendritic density correlates with the *seq* genotype

**Fig 3:**
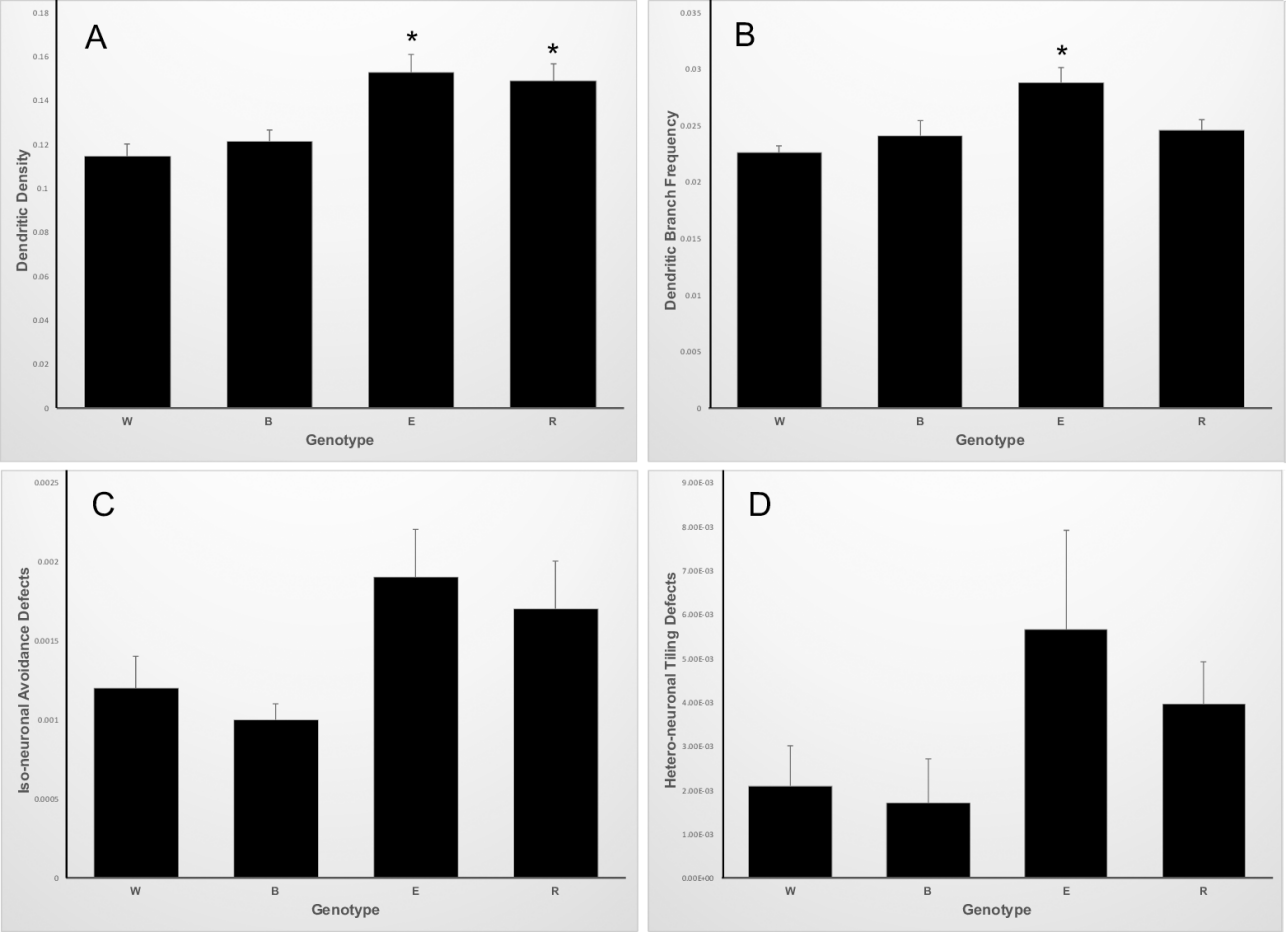
Quantification of ddaC dendrite morphologies in control, mutant and rescued third instar larvae. Genotypes in all panels: W, wildtype larvae, N = 9; B, *trio*-mutant background, N = 8; E, *seq*-enhanced *trio* mutants, N = 9; R, class IV da *trio* expression in the *seq*-enhanced *trio* mutant background, N = 10. Error bars represent the standard error of the means. (A) Dendritic density (dendrite length in μm/μm^2^ surface). ANOVA p-value = 0.0005; E and R are not statistically different from each other, but both are statistically different from both W and B at p<0.05. (B) Dendritic branch frequency (dendritic branches/dendrite length in μm). ANOVA p-value = 0.0022; E is statistically different than W, B and R at p<0.05. (C) Iso-neuronal avoidance defects (iso-neuronal avoidance defects/dendrite length in μm). ANOVA p-value = 0.086. (D) Hetero-neuronal tiling defects (hetero-neuronal tiling defects/μm of path length of interface between dendritic fields). ANOVA p-value = 0.197.

The dendritic density differed amongst these genotypes. Wildtype larvae had a dendritic density of 0.115±0.006 μm/μm^2^, simple *trio* mutant larvae had a dendritic density of 0.121±0.005 μm/mμ^2^, *seq*-enhanced *trio* mutants had 0.153±0.008 μm/μm^2^, and *ppk1*.*9-trio* rescued *seq*-enhanced *trio* mutant larval had 0.149±0.008 μm/μm^2^ (Fig 3A). Thus, animals heterozygous mutant for *seq*^*9*.*17*^, regardless of the status of *trio*, had statistically higher dendritic densities as compared to animals homozygous wildtype for *seq*. This is broadly consistent with previous reports of the *seq* mutant phenotype [16,31], although those reports characterized the *seq* homozygous mutant phenotype, rather than heterozygous mutants. However, this statistically significant difference in dendritic density does not correlate with the ability to undergo the larval-pupal transition, as *seq*-enhanced *trio* mutant larvae generally fail to undergo the larval-pupal transition (Fig 1) while *ppk1*.*9-trio* rescued *seq*-enhanced *trio* mutant larvae generally do undergo the larval-pupal transition (Fig 1).

#### An over-branched dendritic morphology correlates with the failure to undergo the larval-pupal transition

The dendritic branch frequency also differed amongst these genotypes. Wildtype larvae had a dendritic branch frequency of 0.023 ±0.001 branches/μm, simple *trio* mutant larvae had a dendritic branch frequency of 0.024±0.001 branches/μm, *seq*-enhanced *trio* mutants had 0.029±0.001 branches/μm, and *ppk1*.*9-trio* rescued *seq*-enhanced *trio* mutant larvae had 0.025±0.001 branches/μm (Fig 3B). This phenotype correlated with the ability to undergo the larval-pupal transition, as *seq*-enhanced *trio* mutant larvae generally fail to undergo the larval-pupal transition (Fig 1), and that genotype had a statistically higher dendritic branch frequency as compared to all other genotypes (Fig 3B).

#### Iso-neuronal avoidance defects and hetero-neuronal tiling defects did not differ amongst the genotypes

The class IV da neurons’ dendrites effectively enervate the larval epidermis, lacking overlap between different neurons’ arbors (hetero-neuronal tiling) as well as within each neuron’s dendritic trees (iso-neuronal avoidance) [10,32]. The iso-neuronal avoidance defects normalized to total dendrite length in μm did not differ amongst these genotypes. Wildtype larvae had 0.0012±0.0002 defects/μm, simple *trio* mutant larvae had 0.0010±0.0001 defects/μm, *seq*-enhanced *trio* mutants had 0.0019±0.0003 defects/μm, and *ppk1*.*9-trio* rescued larval had 0.0017±0.0003 defects/μm (Fig 3C). Similarly, the hetero-neuronal tiling defects normalized to the path length in μm of the interface between the two dendritic fields did not differ amongst these genotypes. Wildtype larvae had 0.0021±0.0009 defects/μm, simple *trio* mutant larvae had 0.0017±0.0009 defects/μm, *seq*-enhanced *trio* mutants had 0.0056±0.0022 defects/μm, and *ppk1*.*9-trio* rescued larval had 0.0039±0.0009 defects/μm (Fig 3D). Thus, these measures do not correlate with the ability to undergo the larval-pupal transition.

Based on our morphological analysis of the ddaC dendritic fields extending from the ddaC cell bodies to the dorsal midline, neither the density of dendrites, the normalized iso-neuronal avoidance defects, nor the normalized hetero-neuronal tiling defects correlates with the ability to undergo the larval-pupal transition (Fig 3A, 3C and 3D). However, the dendritic branches/dendrite μm measure does correlate with the ability to undergo the larval-pupal transition (Fig 3B), with more highly branched dendrites associating with impaired ability. This correlation is intriguing, and will guide our future work to rigorously test whether more highly branched dendrites are causative in the block of the larval-pupal transition.

Others have investigated the specific role of *trio* in determining class IV da neuron morphology. Expressing RNAi against *trio* in class IV da neurons decreased dendritic branch complexity (decreased total dendritic terminals, decreased total dendritic length, increased average distance per branch and thus decreased overall distribution of ddaC branch order) [17]. Using classical genetics to generate homozygous *trio* mutants has been found to decrease class IV da total dendritic branches and decrease total dendritic lengths [18]. In contrast, our simple *trio* mutant genotype (*trio*^*M89*^ */ trio*^*P1*^) did not show a reduction in dendrite density nor an alteration in dendritic branch frequency (Fig 3A and 3B) presumably because we utilized the *trio* hypomorphic allele *trio*^*P1*^. This hypomorphic allele was originally used to provide a sensitized genetic background in our screen for dominant second-site modifiers of the *trio* mutant phenotype [15]. The increased dendritic branch frequency observed in the larvae that generally failed to undergo the larval-pupal transition (*seq*^*9*.*17*^ */ +; trio*^*M89*^ */ trio*^*P1*^, Fig 3B) is a result of the dosage-sensitive genetic interaction between *seq* and *trio*, as restoring *trio* expression with the GAL4-UAS system brought the dendritic branch frequency back down to levels seen in our wildtype background (Fig 3B).

### Mechanical nociception is separable from the ability to undergo the larval-pupal transition

Mechanical nociception, hypothesized to have evolved in response to small parasitoid wasp oviposition, is functionally dependent on class IV da sensory neuron activity [19–21]. Thus, we tested our larval genotypes for their mechanical nociceptive responses. Wildtype larvae responded to a simulated wasp ovipositor with a 360-degree nocifensive rolling response 83% of the time, simple *trio* mutant larvae responded 87% of the time, *seq*-enhanced *trio* mutants responded 89% of the time, and *ppk1*.*9-trio* rescued *seq*-enhanced *trio* mutant larvae responded 85% of the time (Table 1).

**Table 1:** Mechanical nociception in control, mutant and rescued third instar larvae.

Genotype	N	% Response
wildtype (*trio*^*M89*^ */ +*)	120	83%
*trio*-mutant background *(trio*^*M89*^ */ trio*^*P1*^*)*	101	87%^a^
*seq*-enhanced *trio* mutants (*seq*^*9*.*17*^ */ +; trio*^*M89*^ */ trio*^*P1*^*)*	117	89%^a^
class IV da *trio* expression in the *seq*-enhanced *trio* mutant background (*GAL4-ppk1*.*9*, *UAS-mCD8GFP* / *seq*^*9*.*17*^*; trio*^*M89*^ */ trio*^*P1*^, *UAS-trio*.*B)*	119	85%^a^

^a^ p>0.05 as determined by Chi-squared vs. wildtype

Although both the larval-pupal transition and mechanical nociception behaviors utilize networks involving the class IV da neurons, our results show these two behaviors are separable. The *seq*-enhanced *trio* mutant larvae generally fail to undergo the larval-pupal transition but have fully normal mechanical nociception responses (Fig 1, Table 1). Class IV-specific-*trio* expression generally rescues the larval-pupal transition in these animals with no impact on mechanical nociception (Fig 1, Table 1). Our on-going investigation of this bifurcation of the behavioral responses governed by the class IV da neurons may shed light on the key structure / function relationships in these sensory neurons.

Although we could not characterize class IV da axonal morphologies in this work, these mechanical nociception data add to our interpretation of our *trio*-rescue experiments (Fig 1). Without these data, a trivial explanation for the loss of the larval-pupal transition in the *seq*-enhanced *trio* mutant larvae might be fully disrupted axonal connections of the class IV da sensory neurons, which are then restored by class IV-specific-*trio* expression. However, as the *seq*-enhanced *trio* mutants have fully normal mechanical nociception responses (Table 1), this shows their class IV da sensory neurons are integrated into functional networks with intact axonal connections. While the specific synaptic connections formed by the class IV da neurons may determine their differential functionality between the larval-pupal transition and mechanical nociception responses, the trivial explanation of a loss of larval-pupal response due to overall disrupted axonal connections is excluded.

## Conclusions

In summary, we have shown that *trio* expression in the class IV da sensory neurons rescues the blocked larval-pupal transition phenotype in *seq*-enhanced *trio* mutant larvae. Morphological characterization of the dendritic arbors of a representative class IV da neuron in wildtype, mutant and rescued larvae found a correlation between an over-branched phenotype and the larval-pupae transition block. We have also found that *seq*-enhanced *trio* mutant larvae have normal mechanical nociception responses. As nociception has been previously shown to be dependent on the class IV da sensory neurons, these data demonstrate a bifurcation in two important behavioral responses, the larval-pupal transition and mechanical nociception, influenced by these sensory neurons. All these observations will guide our future research on the relationships between structure and function in class IV da sensory neurons.
